# VDACs: An Outlook on Biochemical Regulation and Function in Animal and Plant Systems

**DOI:** 10.3389/fphys.2021.683920

**Published:** 2021-08-05

**Authors:** Barkha Ravi, Poonam Kanwar, Sibaji K. Sanyal, Malathi Bheri, Girdhar K. Pandey

**Affiliations:** Department of Plant Molecular Biology, University of Delhi, New Delhi, India

**Keywords:** mitochondrial channel, biochemical regulation, phosphorylation, cell signaling, Ca^2+^, reactive oxygen species, cellular homeostasis, CBL–CIPK

## Abstract

The voltage-dependent anion channels (VDACs) are the most abundant proteins present on the outer mitochondrial membrane. They serve a myriad of functions ranging from energy and metabolite exchange to highly debatable roles in apoptosis. Their role in molecular transport puts them on the center stage as communicators between cytoplasmic and mitochondrial signaling events. Beyond their general role as interchangeable pores, members of this family may exhibit specific functions. Even after nearly five decades of their discovery, their role in plant systems is still a new and rapidly emerging field. The information on biochemical regulation of VDACs is limited. Various interacting proteins and post-translational modifications (PTMs) modulate VDAC functions, amongst these, phosphorylation is quite noticeable. In this review, we have tried to give a glimpse of the recent advancements in the biochemical/interactional regulation of plant VDACs. We also cover a critical analysis on the importance of PTMs in the functional regulation of VDACs. Besides, the review also encompasses numerous studies which can identify VDACs as a connecting link between Ca^2+^ and reactive oxygen species signaling in special reference to the plant systems.

## Introduction

Popularly known as the “powerhouse of the cell,” mitochondria have risen from their classical biochemical functionality. The voltage-dependent anion channels (VDACs), first reported by Schein et al. (1976), are the most abundant, multi-functional family of pore-forming proteins present on the outer mitochondrial membrane (OMM; Benz, 1994; Colombini et al., 1996). The general structure of VDAC proteins can be described as a channel formed by 19 β-barrels and an N-terminal that folds in an α-helix, horizontally within the pore (Bayrhuber et al., 2008; Hiller et al., 2008; Ujwal et al., 2008). The studies indicate that the permeability of the pore is majorly regulated by its voltage-dependent conformational state (open state shows slight preference for small anions while its closed state prefer cations) (Colombini, 1980; Benz et al., 1990; Hodge and Colombini, 1997), hence regulating organelle metabolism (Benz et al., 1988, 1990; Ludwig et al., 1988). The determination of VDACs as regulators of the OMM permeability, as well as in major molecular trafficking across the mitochondrial-cytoplasmic interface, revealed a new mitochondrial dimension on how they can regulate inputs according to external cues (Colombini, 1979). Additional discoveries depicted their crucial role in cell signaling, cytoplasmic-mitochondrial communication, aging, cell life, as well as the controversial cell death mechanism (Crompton, 1999; Griffiths, 2000; Colombini, 2004; Baines et al., 2007; Ott et al., 2007). VDACs have been reported from all the eukaryotes studied so far (De Pinto et al., 2003). Three of its isoforms are present in mammals, including humans (Menzel et al., 2009; Raschle et al., 2009; De Pinto et al., 2010), wherein hVDAC3 is accepted as the oldest isoform, and hVDAC1 is the recently evolved isoform (Young et al., 2007). However, their numbers are variable in plants and are generally more as compared to mammals. The *Oryza sativa* and *Nicotiana tabacum* have three VDAC isoforms (Al Bitar et al., 2003; Tateda et al., 2009), *Arabidopsis thaliana* has four VDAC isoforms (Clausen et al., 2004; Tateda et al., 2011), and *Medicago trunculata*, *Lotus japonica*, *Phaseolus vulgaris*, and *Glycine max* contain at least five putative VDACs (Clausen et al., 2004; Wandrey et al., 2004; Saidani et al., 2016). Their variable number in plants indicates that they might have a more diverse role in plants. While protein purification, sequence analysis, and/or structure prediction have already been reported from plants like wheat, rice, pea, pearl millet, maize, and potato (Aljamal et al., 1993; Fischer et al., 1994; Heinss et al., 1994; Elkeles et al., 1995; Geiger et al., 1999; Al Bitar et al., 2003), plant VDACs still require high-resolution structure revelation.

Voltage-dependent anion channel, being one of the major membrane protein in the OMM, can be modulated by factors like chemical compounds and other proteins or cellular metabolites. In this review, we provide an update on the biochemical regulation of VDACs and their impact on the physiological function of VDACs, with a particular emphasis on the plant system. Post-translational modifications (PTMs) are essential mechanisms that diversify protein functions (Lodish, 1981). Several PTMs on VDACs are reported in animal systems and predicted in plant systems (Elkeles et al., 1995; Al Bitar et al., 2003; Martel et al., 2014) and might be responsible for the multi-functionality of these proteins. Therefore, it becomes imperative that we look at their PTMs via phosphorylation and other modes of modification. Lastly, we will tackle the potential role of VDAC in being the focal point through which calcium (Ca^2+^) and reactive oxygen species (ROS) signaling may crosstalk in the cell. This information should help us understand the functional significance of VDACs and explore the possibility that VDACs might have a role in inter-organellar communication for major cellular processes in plants.

## Biochemical and Interactional Regulations Affect VDAC Functioning in Plants

In the following sections, we discuss the various factors that influence the biochemical, structural, and functional aspects of VDACs.

## Chemical Compounds: Interactional Influences I

**FIGURE 1:**
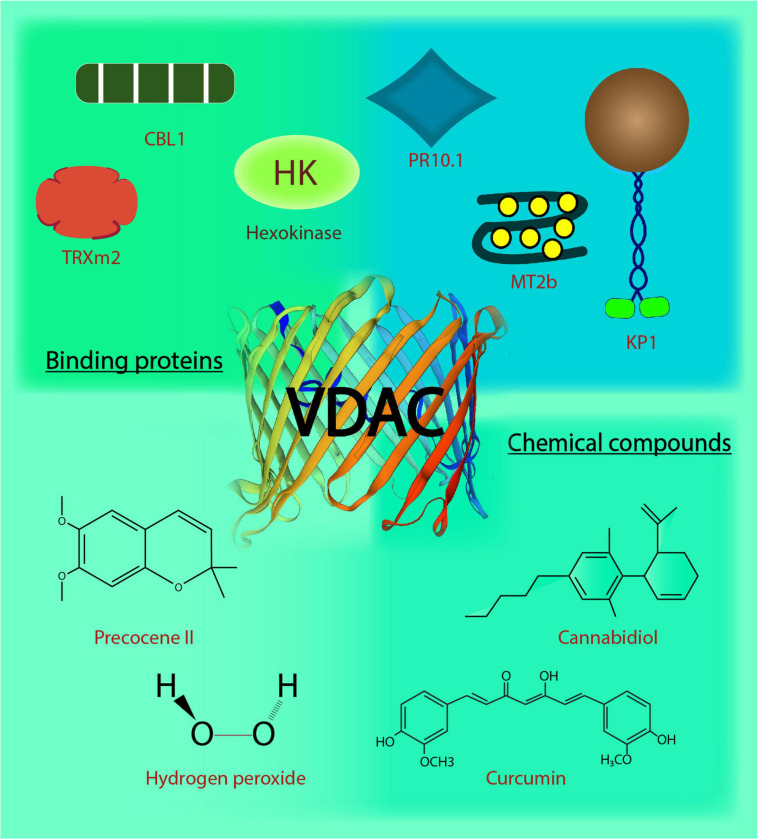
Voltage-dependent anion channels in plants. The biochemical action and interaction of VDACs in plants affects their functional behavior. This image depicts the regulation and interaction of VDACs with binding proteins and chemical compounds. CBL1, Calcineurin B-like protein; TRXm2, chloroplast protein thioredoxin m2; MT2b, metallothionein 2b; H_2_O_2_, hydrogen peroxide; KP1, plant kinesin protein 1; PR10.1, pathogenesis-related 10.1; C_13_H_16_O_3_, precocene II; C_21_H_20_O_6_, curcumin; C_21_H_30_O_2_, cannabidiol.

Natural compounds or extracts from plants have served humankind from ancient times owing to their medicinal properties and diversity. The modulation of VDACs is reported by plant-based compounds (depicted in Figure 1), usually through binding. Curcumin, a pigmented polyphenolic compound extracted from *Curcumin longa*, is known for its anti-cancer properties (Aggarwal et al., 2003). Curcumin modulates hVDAC1 when reconstituted in a lipid bilayer (Tewari D. et al., 2015). Molecular docking and mutational analysis have shown that curcumin interacts with hVDAC1 through N-terminus (Lys 15, Arg 18, and Asp 19) at one end and the amino acid Tyr 198 located in the inner wall of the channel. Closure of hVDAC1 by curcumin might contribute to its known pro-apoptotic property (Tewari D. et al., 2015). An essential oil constituent, precocene II, inhibits the production of tricothecene in *Fusarium graminearum*, the causal agent of fusarium head blight (Yaguchi et al., 2009). This fungal component has been shown to bind the single VDAC in *F. graminearum* through the affinity magnetic bead method. The precocene II is responsible for the increased level of superoxide in mitochondria (Furukawa et al., 2015) postulated due to gate closing. However, its role in trichothecene production needs further validation (Maeda and Ohsato, 2017). Hydrogen peroxide (H_2_O_2_) has been shown to modulate rat brain-VDAC1 through an electrophysiological approach. The conductance of VDAC was shown to increase upon H_2_O_2_ treatment, and its activity was restored by curcumin. The binding site of H_2_O_2_ on the channel identified through *in silico* molecular docking indicates a possibility of direct interaction between VDAC and H_2_O_2_ (Malik and Ghosh, 2020). Leaf extracts (aqueous-methanolic) from *Centella asiatica* (CA) quench ROS during ischemia-reperfusion injury by protecting N2a cells due to antioxidant property. However, leaf extracts from CA failed to prevent IR injury in VDAC1 knocked down-N2a cells, which indicates the involvement of VDACs in the protective effect of CA. This was followed by reconstitution of hVDAC1 in the lipid bilayer membrane, CA treatment resulted in its increased single-channel conductance and stabilized the open state of hVDAC1 (Tewari et al., 2016). This might indicate the modulation of VDACs by CA, aiding its protective effects. A phyto-cannabinoid CBD (cannabidiol) derived from *Cannabis* spp. demonstrates antileukemic activity. They directly binds to hVDAC1 and resulted in decreased channel conductance depicted using western blotting, and single channel conductance in the planar lipid bilayer thereby causing CBD-induced cell death (Rimmerman et al., 2013). *In-silico* hVDAC1-CBD interaction analysis led to the identification of three putative residues at the N-terminus (Thr9, Asp12, and Leu13) and five neighboring pore residues (Val146, Gln157, Gly175, Gln182, and His184). Steric interaction and hydrogen bonds stabilized the binding, with Thr9, Asp12, and His184 contributing most highest in the binding (Olivas-Aguirre et al., 2019). In the follow up report, the effect of CBD and curcumin, along with six other phenolic compounds, was tested for their anticancer activity. Out of these, CBD, curcumin and quercetin (another phenolic compound; Davis et al., 2009) preferred to interact on two specific residues (His184 and Thr9) predicted through *in silico* analysis and displayed cytotoxic effects in human T-ALL Jurket cell lines. CBD and curcumin were found to be the most conspicuous in causing cell death due to mitochondrial Ca^2+^ overload (Olivas-Aguirre et al., 2020). The regulation of human VDACs by plant extracts might indicate a new level of regulation operated by these plant-based metabolites, contributing to protective effects as witnessed in the case of apoptosis and cell death regulation. These plant-based natural compounds have potential to serve as anti-cancer drugs targeting mitochondria through VDACs. But, these prediction based on the above-mentioned studies lacks clinical potential viability and they need further exploration. Overall, the *in vitro* findings with reconstituted VDACs can help us infer better on the effect of plant extracts on VDACs and mitochondrial physiology in general. Moreover, their *in vivo* characterization will shed light on their degree of utilization. The identification of these candidate regulators from plants holds great therapeutic relevance.

## Proteins: Interactional Influences II

Voltage-dependent anion channel’s interaction with many cytosolic proteins makes it a nexus for multiple cell signaling events. Interestingly, in plants, the reports on VDACs and their interacting partners (depicted in Figure 1) indicate that VDAC (and the particular partner) have a role in responding to abiotic and biotic stress, inter-organelle connection, metabolite flux at the cytosolic-mitochondrial interface, in addition to VDAC’s probable role in programmed cell death. To begin with the role in stress signaling mechanism, a type 2 metallothioneins (MTs: small cysteine-rich, metal-binding proteins), have been shown to directly interact with AtVDAC3 in Arabidopsis through yeast two-hybrid (Y2H) and bimolecular fluorescence complementation (BiFC) assays. The *AtMT2b* overexpression plants exhibited increased salt tolerance, and AtMT2b may act as a negative regulator of AtVDAC3 (Zhang et al., 2019). Further, plant-specific calcineurin B-like proteins (CBL) are one of the major Ca^2+^ sensors that integrate Ca^2+^ signaling to various abiotic stresses in plants (Pandey, 2008; Sanyal et al., 2015; Sanyal et al., 2016; Sanyal et al., 2020). AtVDAC1 in Arabidopsis physically interacts with AtCBL1 *in vitro*. Together, they regulate cold stress responses during plant development and seed germination (Li et al., 2013). This may also connect Ca^2+^ and ROS signaling pathways, extensively reviewed in the animal systems and discussed later in this review (Yan et al., 2006; Görlach et al., 2015; Feno et al., 2019). Recent discovery on mitochondrial regulation through interaction between chloroplast protein, thioredoxin m2 (AtTrx m2), and AtVDAC3, emphasizes an inter-organellar communication (Zhang et al., 2015). Y2H and pull-down assays have confirmed the physical interaction between the two, and BiFC assay has located the interaction on the mitochondria. This may act as another partner of VDAC3 for regulating ROS signaling and salt stress responses in plants (Zhang et al., 2015). VDAC3 from *Vitis piasezkii*’ Liuba-8 was screened as an interacting partner of pathogenesis-related PR10.1 obtained from *Vitis pseudoreticulata*’ Baihe-35-1 in Y2H library screening. The interaction was further confirmed through immunoprecipitation assays; together they function in imparting cell death mediated defense response to downy mildew disease caused by *Plasmopara viticola*, a biotrophic parasite in grapevine (Ma et al., 2018). The finding substantiates the functional role of plant VDACs in pathogen defense (Lee et al., 2009; Tateda et al., 2009). Several animal kinesins such as those from mouse cells, KIF1B as well as KIF5B and *Drosophila melanogaster*, KLP67A kinesin are involved in the movement of mitochondria (Nangaku et al., 1994; Pereira et al., 1997; Tanaka et al., 1998). A plant-specific kinesin, KP1, is also found to interact with plant mitochondrial AtVDAC3 in Arabidopsis. AtKP1 interacts with AtVDAC3 via its tail domain and regulates respiration during seed germination at low temperatures in plants (Yang et al., 2011). This interaction between microtubule motor protein and mitochondrial channel indicates possibilities of involvement of plant kinesins in controlling the movement of mitochondria, which requires further investigation.

Mitochondria are the hub of aerobic oxidation in eukaryotic cells. The glycolytic enzymes associate dynamically with mitochondria and support respiration. Cytoskeletal protein tubulin can induce a reduced respiration rate by reversible blocking of VDAC, as observed through patch-clamp and planar lipid bilayer technique in animal and fungal systems (Rostovtseva et al., 2008). The involvement of VDACs in the regulation of respiration is reported in plants as well. Glycolytic enzymes, such as aldolases, interact strongly with AtVDAC protein and the latter may anchor the enzyme at the mitochondrial surface, thus facilitating substrate channeling (Graham et al., 2007). These findings are important in the study of the regulation of metabolic flux and involvement of VDACs in the plant metabolic networks. Additionally, the interaction of VDACs by glycolytic enzyme, hexokinases through *in vitro* and *in vivo* studies is a well-studied concept in animals (Abu-Hamad et al., 2008; Galluzzi et al., 2008; Pastorino and Hoek, 2008) and is projected as a strategy to aid conventional chemotherapeutics (Pastorino et al., 2002; Abu-Hamad et al., 2008). Mitochondrial hexokinase modulates the function of endogenous VDAC in tobacco bright yellow cell-2 (BY2 cells) as well as heterologous expressed OsVDAC4 in *N. benthamiana* leaves. Their co-expression limits their toxicity (independently overexpressing both the proteins causes toxicity in plants and cell death in BY2 cell lines), resulting in healthy cells and leaves in plants. The expression ratio of VDAC–hexokinase and their interactions are essential in cell death pathways in plants (Kim et al., 2006; Godbole et al., 2013). The heterologous expression indeed provides a clue on the importance of VDAC–hexokinase interaction; however, the functional validation of this entire pathway in the endogenous rice system itself can conclude better about its actual functionality.

## Post-Translational Modifications: Regulation of VDAC Function

Reversible PTMs affect VDACs and their interaction with other proteins. VDACs are modulated post-translationally by phosphorylation, acetylation, GlcNAcylation, oxidative post-translation modifications (Ox-PTMs), and ubiquitination. These modifications on VDACs will be discussed in the following sections.

## Regulation Through Phosphorylation

Voltage-dependent anion channels undergo phosphorylation for modulation of their channel properties. To understand the biological significance of the phosphorylation event(s) in VDAC, it is necessary to identify the phosphorylation sites in VDACs, the protein mediating the phosphorylation, the physiological stimulus mediating the phosphorylation and the resultant change in VDAC (in particular) and physiology in general. *In vitro* and *in silico* analyses revealed many phosphorylation sites in isoforms of VDAC, which can play an important role in studying the function of VDAC in the near future. If we look into the studies performed till date to understand the phosphorylation events of VDAC, we can divide them into three main classes: (a) studies that proved VDAC can be phosphorylated which alters the channel properties, (b) studies where the whole proteome was examined to understand the PTM and (c) where the specific study on VDAC phosphorylation site was performed, followed by identification and validation.

The first group majorly consists of reports from the Ghosh laboratory (Bera et al., 1995; Bera and Ghosh, 2001; Banerjee and Ghosh, 2006; Gupta and Ghosh, 2017). They have used VDAC (not isoform-specific) from either rat liver (Bera et al., 1995; Bera and Ghosh, 2001) or rat brain (Banerjee and Ghosh, 2006; Gupta and Ghosh, 2017). This was followed by assessing the phosphorylation against protein kinase A (PKA) (Bera et al., 1995; Bera and Ghosh, 2001), the effect of Bax and tBid binding on VDAC (Banerjee and Ghosh, 2006) and PKA mediated phosphorylation and c-Jun N-terminal kinase-3 (JNK3) (Gupta and Ghosh, 2017). They have identified PKA and JNK3 mediated phosphorylation of VDAC through *in vitro* kinase assays and followed it up with investigation of the changes in channel dynamics of VDAC due to phosphorylation in real time, using reconstitution in lipid membrane bilayer (Bera et al., 1995; Bera and Ghosh, 2001; Banerjee and Ghosh, 2006; Gupta and Ghosh, 2017). Their protocol, though, has given important insights into the phosphorylation of VDAC and the resultant change in the gating properties but lacks the information on the actual VDAC isoform being phosphorylated and also the phosphorylation site(s). Another similar study was reported by Baines and colleagues, where they analyzed the *in vitro* phosphorylation of mouse VDAC1 by protein kinase C (PKC). These studies gave some critical shreds of evidence on the PKC and VDAC interaction and probable regulation of mitochondrial permeability transition pore by PKC, but in terms of phosphorylation, the information did not yield more knowledge beyond just the event.

The second group of studies provided an improvement from the first group by indicating the exact site of phosphorylation in VDAC (and also the particular isoform) under a certain stimulus (or a disease). Tyr phosphorylation of pig VDAC1 and VDAC2 were reported using immunoblotting followed by mass spectrometry (MS) under hypoxia, with no specific site identified (Liberatori et al., 2004). Using anti-Phospho-Tyr antibodies, Schwertz et al. (2007) showed that in rabbits, VDAC1 underwent Tyr phosphorylation during myocardial ischemia by p38 MAPK. Ballif et al. (2008) used MS on peptides extracted from mouse brain and identified Tyr phosphorylation on VDAC1 (Tyr80 and Tyr208), VDAC2 (Tyr207), and VDAC3 (Tyr49). Another study performed on HeLa cells stimulated by epidermal growth factor (EGF) identified phosphor-sites [(Ser101, Ser102, and Ser 104) and Thr 107] in VDAC1 and (Ser115 and Thr118) VDAC2 by MS (Olsen et al., 2006). All three isoforms of rat VDACs were phosphorylated at one or more sites, presumably without any treatment. The study identified Ser12 and Ser136 in VDAC1, Tyr237 in VDAC2, and Thr33 and Ser241 in VDAC3. These results were confirmed by MS (Distler et al., 2006, 2007). Two different groups reported Ser117 as a possible phosphorylation site in mouse VDAC1 using different tissues, again presumably without treatment (Lee et al., 2007; Munton et al., 2007). Tewari S. G. et al. (2015) have used a phospho-mimetic VDACSer137Glu in their studies and have shown that it affects the channel properties of rat VDAC1 (the probable isoform). A quick check of the rat VDAC1 sequence in the UniProt database (Q9Z2L0) indicates that the Ser is at position 137 (at 136 position there is a Pro). Therefore, we believe that it is the same Ser reported by Distler and colleagues (and also claimed by Tewari and colleagues). These studies were advancements on the first group as they yielded exact information on the VDAC isoforms and phosphorylation sites, and the last report indicates that there is merit in examining them more carefully.

Addressing the third and final group, that looks into the VDAC phosphorylation sites followed by validation of experimental results. There are three main candidates here: (1) the phosphorylation of VDAC by glycogen synthase kinase-3 (GSK-3), (2) the phosphorylation of VDAC during endostatin treatment, and (3) phosphorylation of VDAC by NIMA-related protein kinase 1 (Nek1). VDAC can be phosphorylated by Akt and GSK-3 (proved from rat heart, mouse liver and humans VDAC) (Pastorino et al., 2005; Das et al., 2008; Sheldon et al., 2011; Martel et al., 2014). Pastorino et al. (2005) used VDAC precipitated from HeLa cells (different stimulus applied to perturb GSK-3 expression) using a mouse anti-VDAC antibody and then assessed the phosphorylation status using an anti-Phosphothreonine antibody. They further created a phosphor-mutant VDAC3Thr51Ala (from the consensus sequence for GSK-3 phosphorylation). An *in vitro kinase* assay showed that this particular variant was not phosphorylated by GSK-3 (Pastorino et al., 2005). By analyzing the mouse VDAC sequences provided in UniProt, we posit that the VDAC used in this study was either hVDAC1 (P21796) or hVDAC3 (Q9Y277) as they both have Thr at position 51. The phosphorylation of VDAC by GSK-3 at Thr residues were also validated in mice and human models by Martel et al. (2013) using both antibody (anti-Phosphothreonine) based approaches and *in vitro* kinase assay. This particular study used MS only to identify the VDAC isoform and not the phosphorylation site(s). But there are more Thr residues available (in humans, mice, and rats) that could be phosphorylated and need further validation. Nevertheless, Thr51 in human VDAC1 (or VDAC3) is a target of GSK-3 for modulating the channel properties. Yuan et al. (2008) showed that in endostatin-treated human cell line, VDAC1 could be immunoprecipitated by using a rabbit VDAC1 antibody, and its phosphorylation was detected on western blotting by probing with anti-Phosphoserine antibody. They created phosphor-mutant Ser12Ala and Ser103Ala, which showed reduced human VDAC1 accumulation after endostatin treatment, indicating that these sites are the probable phosphorylation sites. Hexokinase II, endostatin, PKC, and GSK-3 modulates VDAC function, however, it was hypothesized that either PKC or GSK-3 are the potential kinases regulating the phosphorylation of human VDAC1 in this case. Although there is no concrete proof of the identity of the kinases, this does question the earlier results, which had shown GSK-3 phosphorylating primarily Thr residues. Chen et al. (2009, 2010) reported that Nek1 interacts and phosphorylates human VDAC1 on Ser193 (by creating a phosphor mutant VDAC1Ser193Ala) residue using *in vitro* kinase assays. The Nek1 is regulated by TKL1 (Tousled-like kinase), which is also involved in chromatin assembly and DNA repair mechanism (Sunavala-Dossabhoy and De Benedetti, 2009). On investigating the entire TLK1-Nek1-VDAC1 module, VDAC1 was found to be a key link between mitochondria-mediated apoptosis and irreparable DNA damages (Singh et al., 2020).

We provide an interesting anecdote where it was shown that a kinase partner could modulate VDAC without phosphorylation. A Raf family Ser/Thr kinase, C-Raf, interacts and is targeted to OMM by Bcl2 protein in a kinase-substrate independent manner and is shown to suppress apoptosis in hemopoietic cell lines (Wang et al., 1994, 1996). However, a Bcl-2 independent mechanism controlled by C-Raf is also reported where C-Raf targeted to OMM forms a complex with human VDAC1 observed in co-immunoprecipitation studies. No phosphorylation was reported for this interaction (in both *in vivo* and *in vitro* experiments) (Le Mellay et al., 2002).

The phosphorylation sites detailed in the above paragraphs (and summarized in Table 1) put forward a fundamental question regarding the identity of the “phosphorylation switches” that can be utilized for future studies to modulate the VDAC for *in vivo* research. Although VDACs have similar sequences, there are abundant proofs that phosphorylation sites can differ based on species and kinase involved. We believe that the best approach to deal with this problem is -after the identification of a kinase partner of VDAC, an *in vitro* kinase assay is to be followed to first answer the question of phosphorylation-based regulation. This should be then followed by identification of sites by MS following either perturbing the organism with the stimulus that is being investigated or alternatively, by MS after an *in vitro* kinase assay. The results obtained from the MS analysis should be used to create either phospho-mimic or phosphor-mutant, and then one can proceed to analyze the channel conductance of these variants. These approaches should give us better results for the identification of actual site(s). Distressfully, the data on plant VDAC phosphorylation is still lacking.

**TABLE 1:** The table shows the VDAC isoforms phosphorylated by candidate protein kinases detected by mass spectrometry (MS), site directed mutagenesis (SDM) and predicted sites with their functional consequences.

Isoform	Regulation	Phosphorylation site	Mode of site detection	Organism/organ interaction found in	Protein kinase	Functional relevance
VDAC1	Phosphorylation	S^13^, S^137^, S^234^	Predicted (Wang C. et al., 2020)	Rat heart	PKCε	Cardio-protection (Baines et al., 2003)
VDAC1	Interaction	S^12^, S^103^	Experiment	Human microvascular endothelial cells	ES	Endostatin-induced endothelial cell apoptosis (Yuan et al., 2008)
VDAC1	Interaction	Not detected	None	Rat liver	C-Raf	Apoptotic suppression (Le Mellay et al., 2002)
VDAC1	Phosphorylation	S^45^	Predicted (Wang C. et al., 2020)	Rat liver	PKA	Cytochrome mediated cell death (Banerjee and Ghosh, 2006)
VDAC1	Phosphorylation	S^193^	Experiment	Human cell lines	Nek1	Mitochondrial Dysfunction and apoptosis (Chen et al., 2009)
VDAC1	Phosphorylation	S^101^*, S^102^*, S^104^*, T^107^*, S^117^, T^208^	Experiment	Rat liver, Human cell lines*	None	Not defined
VDAC1	Phosphorylation	S^136^	Experiment	Rat liver	PKC	Not defined
VDAC1	Phosphorylation	S^45^, S^186^	Predicted (Wang C. et al., 2020)	Rabbit	p38 MAPK	Ischemia/reperfusion injury (Schwertz et al., 2007)
VDAC2	Phosphorylation	T^51^	Experiment	Rat heart	GSK3	Ischemia/reperfusion injury (Pastorino et al., 2005; Das et al., 2008)
VDAC2	Phosphorylation	S^115^, T^118^	Experiment	Human cell lines	None	Not defined
VDAC2	Phosphorylation	T^237^	Experiment	Rat liver	INSR (Distler et al., 2007)	Not defined
VDAC2	Phosphorylation	T^207^	Experiment	Rat brain	None	Not defined
VDAC3	Phosphorylation	S^241^	Experiment	Rat liver	None	Not defined
VDAC3	Phosphorylation	T^33^	Experiment	Rat liver	None	Not defined
VDAC3	Phosphorylation	S^137^	Experiment	Rat liver	None	Gating kinetics (Tewari S. G. et al., 2015)
VDAC3	Phosphorylation	T^49^	Experiment	Rat brain	None	Not defined
VDAC	Phosphorylation	T^6^, S^104^, S^137^	Predicted (Wang C. et al., 2020)	Rat brain	JNK3	Gating process
VDAC1	Phosphorylation	S^44^, T^45^, S^103^, S^233^, S^264^	Predicted (Elkeles et al., 1995)	Wheat (*Triticum aestivum*)	PKC	Not defined
VDAC2	Phosphorylation	S^41^, T^42^, T^223^, S^262^, S^270^	Predicted (Elkeles et al., 1995)	Wheat (*Triticum aestivum*)	PKC	Not defined
VDAC3	Phosphorylation	T^44^, S^102^, S^225^, S^264^	Predicted (Elkeles et al., 1995)	Wheat (*Triticum aestivum*)	PKC	Not defined
VDAC1	Phosphorylation	T^81^, T^156^, S^206^	Predicted (Elkeles et al., 1995)	Wheat (*Triticum aestivum*)	Creatine kinase	Not defined
VDAC2	Phosphorylation	T^78^, T^164^, S^204^, T^214^, T^252^	Predicted (Elkeles et al., 1995)	Wheat (*Triticum aestivum*)	Creatine kinase	Not defined
VDAC3	Phosphorylation	S^71^, T^80^, T^164^	Predicted (Elkeles et al., 1995)	Wheat (*Triticum aestivum*)	Creatine kinase	Not defined
VDAC1, VDAC2, VDAC3	Phosphorylation	T^51^, S^109^, S^269^	Predicted (Al Bitar et al., 2003)	Rice (*Oryza sativa*)	PKC	Not defined
VDAC1, VDAC2, VDAC3	Phosphorylation	T^87^, T^170^, S^209^	Predicted (Al Bitar et al., 2003)	Rice (*Oryza sativa*)	Casein Kinase II	Not defined

PKC, protein kinase C; ES, endostatin; C-Raf, Raf family Ser/Thr kinase; PKA, protein kinase A; Nek1, NIMA-related protein kinase 1; p38 MAPK, p38 MAP kinase; GSK3, glycogen synthase kinase; INSR, insulin receptor; JNK3, c-Jun N-terminal kinase-3. * Represents Human cell lines

## Regulation Through Alternative Routes of Modification

Other than phosphorylation, regulation of VDACs necessitates comprehension of its alternative modes of PTM. Its regulation has also been observed via nitrosylation/nitrosation, acetylation, oxidation, and interaction with ubiquitin proteins. *In vivo* PTMs by nitrosylation of VDACs have not been directly reported, however, VDAC activity is known to be affected by Nitrous Oxide (NO) (Cheng et al., 2011). Moreover, *in vitro* nitrosation using PAPA NON-Oate (PPN) at 25 μM showed that the conductance is reduced and the dwell time of rat VDAC is increased in the closed state. Also, at PPN100, the conductance was reported to be half of that is present in wild type (wt) (Tewari S. G. et al., 2015). The modification of VDACs induces significant changes in gating kinetics, which might affect mitochondrial (dys)function. The second type of PTM observed in VDACs is O-GlcNAcylation. O-GlcNAcylation affects protein-protein interactions, activity, stability, and expression (Dias and Hart, 2007). VDACs show an increase in O-GlcNAcylation in rats that exhibit low running capacity. This modification might increase mitochondrial stability (Darley-Usmar et al., 2012; Johnsen et al., 2013). Interferon-induced protein with tetratricopeptide repeats 3 (IFIT3) is an interferon-stimulated through JAK/STAT signaling pathway during pancreatic ductal adenocarcinoma (PDAC) (Platanias, 2005; Neuzillet et al., 2015). It directly interacts and regulates VDAC2 through O-GlcNAcylation, which protects PDAC cells from chemotherapy-induced apoptosis (Wang Z. et al., 2020). The modification of VDACs by GlcNAcylation serves to increase mitochondrial stability. Protein modification through acetylation is also crucial in cellular function, though its biological significance is reported for limited substrates (Polevoda et al., 1999; Perrier et al., 2005; Caesar et al., 2006). It either occurs co-translationally at N-terminal residues or post-translationally on lysine (Lys) residue. Acetylation is reported at alanine, Ala 283 residue located at the N-terminal of VDAC1 from mature rat mitochondria, although its significance is not reported yet (Distler et al., 2007). However, they are suggested to function in protein-protein interaction, accumulation of mature protein in target organelles, and general protection of protein from degradation (Hershko et al., 1984; Manning and Manning, 2001; Pesaresi et al., 2003). Ox-PTMs, another type of PTMs reported in VDACs, is an emerging field, and it has broadened our understanding of redox regulation (Stipanuk et al., 2009; Ryan et al., 2014; Shakir et al., 2017). Cysteine (Cys) PTMs in VDAC isoforms have been recently determined using MS (Reina et al., 2020). There are two, nine, and six Cys residues in VDAC1, VDAC2, and VDAC3, respectively. Most of them are localized to facilitate their exposure to IMS (inter-membrane space). Detailed profiling of redox state of Cys and Met of rat VDAC3 has been reported. The evolutionary conservation of Cys modification in the three isoforms is observed in the rat and human VDACs (Saletti et al., 2017, 2018; Pittalà et al., 2020). A conserved oxidative status of Cys residues in rat and human VDAC1 is detected as trioxidized form (Cys127) and the reduced and carboxyamidomethylated form (Cys232) (Pittalà et al., 2020). However, in hVDAC1, there is no change in activity through the Ox-PTMs of the sulfhydryl groups. Cross-linking experiments deny the involvement of intermolecular S-S bridge leading to oligomerization (Aram et al., 2010; Teijido et al., 2012). In the case of VDAC2, the N-terminal Cys 8, 13, and 227 residues located on loop exposed to cytosol were reduced; Cys 47, 76, 103, and 210 residues, localized toward IMS were partially oxidized; and the Cys127 residue localized toward lipid environment was fully tri-oxidized (Pittalà et al., 2020). hVDAC3 contains six Cys2, 8, 36, 65, 122, and 229 residues. MS analyses showed that the Cys 2, 8, 122, and 229 residues were reduced, N-terminal Cys residue 2 was acetylated, residue 36 and 65 were reduced, and tri-oxidized to sulfonic acid (Saletti et al., 2018; Pittalà et al., 2020). Functional significance of the modifications in VDAC3 is reported for some of the residues. Electrophysiology and the ability to revert the growth phenotype of the yeast mutant, Δ*por1*, is strongly affected upon the deletion of Cys residues from engineered human VDAC3 (Okazaki et al., 2015; Reina et al., 2016). Cys 2, 8, and 122 residues seem to be more important for protein function; mutating them or a simultaneous mutation of any of these three Cys to Ala leads to restoration of large pores and the Δ*por1* phenotype (Reina et al., 2016; Queralt-Martín et al., 2020). Cys modifications vary with isoforms as well as their position on VDAC channel. They could function in ROS signaling in mitochondria and this link needs to be explored further. We do not know the significance of the Cys residues in plants. The Arabidopsis VDACs have lesser (or none) Cys residues, and this may indicate an evolutionary change in the way plant VDACs function (Sanyal et al., 2020). Overexpression of *VDAC3* is related to microglial I/R injury (Yao et al., 2018). VDACs exhibit temperature-sensitivity on activation and may contribute to hypothermic neuroprotection against oxygen-glucose deprivation/recovery (OGD/R) (Imada et al., 2010). It is facilitated by interaction between VDAC3 and ubiquitin. The extent of VDAC3 ubiquitination was found directly proportional to hypothermia duration, which may act as an endogenous protective pathway in hypothermia (Zhao et al., 2020). After many decades of research on VDACs, the molecular understanding of their PTM is still limited. The reports on the PTMs and regulation of VDACs are mostly limited to the identification of site and modes of regulation. Though several phosphorylation sites are present on VDACs, only a few are functionally characterized. More information on acetylation, GlcNAcylation, Ox-PTMs and ubiquitination is needed, particularly their functional characterization. Although phosphorylation has been studied more than other modifications, the mere identification of phosphorylation sites is not enough to relate to the functional attributes. Their biological significance needs to be examined critically to enable their implementation. Candidate interactors that regulate VDAC activity have immense therapeutic potential. In comparison, PTMs in the plant VDAC are still an emerging concept and need to be investigated substantially by the plant science community.

## VDACs Connect Ca^2+^ and ROS Signaling

Ca^2+^ is an important second messenger and plays a crucial role in signaling in all eukaryotes (Poovaiah et al., 1987; Clapham, 1995; Rudd and Franklin-Tong, 1999, 2001; Sanders et al., 1999; Trewavas, 1999; Harper, 2001; Pandey, 2008; Kudla et al., 2018; Tang et al., 2020). In a non-excitable cell, Ca^2+^ flux occurs between cytosol, plasma membrane (PM), and endoplasmic reticulum (ER). However, organelles such as mitochondria can also regulate Ca^2+^ signals (Patel and Muallem, 2011; Stael et al., 2012; Wagner et al., 2016; Costa et al., 2018). They are known to be critical regulators of cellular Ca^2+^ homeostasis. They can accumulate ions, including Ca^2+^ (up to several hundred times of initial Ca^2+^ concentration), from the suspended medium during electron transport (Rossi and Lehninger, 1963). The Ca^2+^ sequestration is an energy-dependent process (Gunter et al., 1994). Mitochondrial Ca^2+^ uptake and Ca^2+^ signals play a key role in cellular processes ranging from energy metabolism to cell death. Plant mitochondrial functions range beyond the passive storage/buffering of cytosolic Ca^2+^; they generate unique Ca^2+^ signatures in response to the external stimuli. Cytosolic and mitochondrial Ca^2+^ signatures are generated on the perception of stimuli, particularly under stress. Also, similar to cytosolic Ca^2+^ signatures, mitochondrial Ca^2+^ signatures are differentially sensitive to stimuli. Exposure to cold, touch, and osmotic stress results in very similar temporal kinetics of cytoplasmic free Ca^2+^ concentration, [Ca^2+^]_c_ and mitochondrial free Ca^2+^ concentration, [Ca^2+^]_*m*_. This reflects its role in buffering the [Ca^2+^]_c_, with the stimuli such as oxidative stress and touch showing an independent Ca^2+^ regulation of mitochondria in addition to cytosolic signatures (Logan and Knight, 2003). Ca^2+^ concentration in the cytosol and mitochondria is regulated by utilizing Ca^2+^ transporters across the IMM. However, to facilitate Ca^2+^ flux across the organelle, it needs to bypass the OMM. This regulation requires certain Ca^2+^ transport/regulating system on OMM. The regulating mechanism/molecular components responsible for this flux of Ca^2+^ from mitochondria may help understand the process and utilize it further. VDAC located on OMM shows permeability to Ca^2+^, thus, acting as an important players in Ca^2+^ flux. VDAC is a candidate regulator that manages Ca^2+^ flow in and out of mitochondria (Wagner et al., 2016). It will be interesting to identify other regulatory components in such regulation and examine if it merges with cytosolic Ca^2+^ signaling for better stress adaptation in cells. However, the relationship between VDAC and Ca^2+^ is entrenched beyond their role in the Ca^2+^ movement. The role of VDACs regulating Ca^2+^ signaling in plants is beginning to emerge. Under unfavorable conditions, mitochondria connect and form a network with the rest of the cell to maintain cellular homeostasis (Zemirli et al., 2018). Ca^2+^ signaling is one of the modes of regulation. The physical interaction between AtVDAC1 and CBL1 is one such example, as described earlier. The expression profile of both *AtVDAC1* and *CBL1* indicates the role of this module in cold stress during seed germination (Li et al., 2013). Salt overly sensitive (SOS) pathway mediates Ca^2+^ based cellular signaling under salt stress (Ji et al., 2013). VDAC protein levels were elevated in response to short-term salinity exposure in maize roots (Zörb et al., 2010). Similarly, VDAC2 in plants may also be connected with Ca^2+^ signaling. AtVDAC2 might participate in stress response pathways, as the expression level of *SOS* genes changes with the expression level of *VDAC2* in qRT-PCR studies (Wen et al., 2011, 2014; Liu et al., 2015). Though a direct involvement of VDACs is not reported in plants, however evidence does show their involvement in Ca^2+^ signaling. This indirect Ca^2+^ involvement may be an additional mode of stringent regulation in cellular signaling. Since research based on plant VDAC-Ca^2+^ regulation is bound to flourish in future, it is important to optimize protocols for quantification of Ca^2+^ levels. The technical difficulties in measuring mitochondrial Ca^2+^ levels have been overcome through the development of several *in vivo* Ca^2+^ monitoring methods. The developments in the measurement of mitochondrial Ca^2+^ levels have been covered in several studies (Pozzan and Rudolf, 2009; Jean-Quartier et al., 2012; McKenzie et al., 2017; Fernandez-Sanz et al., 2019). Therefore, the *in vivo* dynamics of these Ca^2+^ signaling components and VDACs can shed more light on the real-time cellular Ca^2+^ status in unfavorable conditions.

VDAC mediated cyto-mitochondrial signaling as well as VDAC-mediated inter-organellar communication through Ca^2+^ in cellular metabolism and survival are vital to our understanding. Mitochondria can act as high capacity Ca^2+^ buffers that determine cytosolic [Ca^2+^] transients through regulation of the kinetic characteristics of Ca^2+^ channels or prevention of Ca^2+^ diffusion from the location of open channels. They may be critically involved in constraining Ca^2+^ signals in spatial terms to specific cellular domains (Rizzuto et al., 2012). The presence of Ca^2+^ hot spots on the mitochondrial surface in the regions closely apposed to Ca^2+^ channels localized on the PM or ER, contribute to the fast and high levels of Ca^2+^ uptake by mitochondria as observed in live cells (De Stefani et al., 2016). The proximity between the mitochondrial and ER membranes favors Ca^2+^ exchanges between them. Their entwined endomembrane network may regulate intracellular Ca^2+^ signaling via VDACs. The transmission of Ca^2+^ signals between the two was imaged through Ca^2+^ sensitive GFPs and aequorin probes. The Ca^2+^ release from ER was coordinated with Ca^2+^ uptake from mitochondria via VDAC (Rapizzi et al., 2002). The coupling of the two organelles can be chaperone-mediated, which directly enhances the accumulation of Ca^2+^ in mitochondria. Glucose regulated protein 75, a cytosolic chaperone homologous to HSP70, physically connects VDAC1 (on OMM) to Inositol 1,4,5-triphosphate receptor (IP_3_R: on ER) and is a determining factor for the interaction as its absence abolished the stimulatory effect. This direct enhancement of Ca^2+^ accumulation in mitochondria forms a molecular bridge (Szabadkai et al., 2006). Co-immunoprecipitation assays further indicate the complex formation of IP_3_ receptor and VDAC1, regulated by apoptotic stimuli. VDAC1 silencing impaired the transfer of apoptotic Ca^2+^ signals selectively (De Stefani et al., 2012). Together, these reports demonstrate the regulation of inter-organellar communication through Ca^2+^ signaling, and VDACs constitute a significant part of this machinery. Ca^2+^ mediated inter-organellar connections in plants are beginning to emerge. This has been discussed in several reviews (Stael et al., 2012; Kmiecik et al., 2016; Himschoot et al., 2017; Costa et al., 2018; Liu and Li, 2019; Navazio et al., 2020).

**FIGURE 2:**
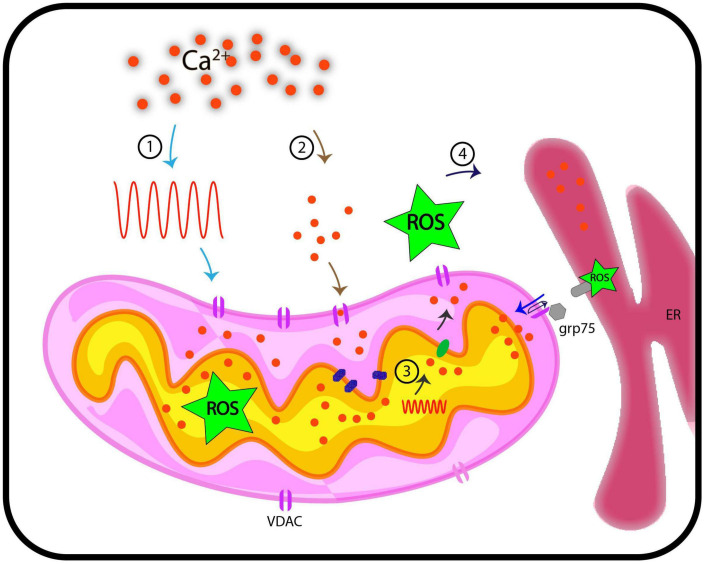
Representation of functional implications of VDACs, Ca^2+^, and ROS in cellular signaling. This hypothetical model traces pathways through which VDAC may regulate Ca^2+^ and ROS signaling in plants. We discuss here four possible routes that we currently posit are VDAC mediated connection between Ca^2+^ and ROS signaling. (1) When cytosolic Ca^2+^ level increases on perception of stimuli (generation of Ca^2+^ signature), these signals are transduced downstream, where they influence the activity of VDACs. This might results in increased level of ROS in mitochondria. (2) VDAC serves passively to regulate Ca^2+^ levels in mitochondria. (3) Stimuli such as oxidative stress (inclusive of increased ROS level) and touch response results in generation of Ca^2+^ signature in mitochondria, independent of cytosolic Ca^2+^ signature. (4) VDAC can be a candidate protein that leads to flux of Ca^2+^/ROS from inter-membrane space to cytosol. Further, VDACs are known to connect ER based Ca^2+^ signaling and mitochondria through Ca^2+^ and ROS molecules and this may depict another possible mechanism of how VDAC connects Ca^2+^ and ROS. These hypothetical routes may (or may not) be connected. ER, endoplasmic reticulum; ROS, reactive oxygen species; grp75, glucose related protein 75; Ca^2+^, calcium; VDAC, voltage-dependent anion channel.

Antioxidant systems in mitochondria regulate the maintenance of ROS at physiological levels through mitochondrial metabolism. ROS imbalances in mitochondria, either through overproduction or impaired antioxidant system, lead to mitochondrial dysfunction and induction of the apoptotic cascade. Such ROS imbalances in pathological conditions exhibit a Ca^2+^ overload in mitochondria (Feno et al., 2019). Since mitochondrial ROS can act as a signaling molecule and not just be a by-product of oxidation reaction during ATP production, it is interesting to look at its signaling aspect (Collins et al., 2012; Sena and Chandel, 2012; Sullivan and Chandel, 2014). Mitochondrial Ca^2+^ uptake impinges on enzymes of the TCA cycle and the activity of the electron transport chain (ETC), generating ROS signals under physiological conditions depending on tissue specificity. This signaling aspect depends on [Ca^2+^] threshold, which, when overcome, results in detrimental mROS levels, compromising mitochondrial bioenergetics and cellular functioning (Brookes et al., 2004; Görlach et al., 2015; Feno et al., 2019). The reciprocal interactions between the major signaling components, Ca^2+^ and ROS, regulating each other, form a “feed-forward, self-amplified loop.” The resultant oxidative damage can be more than that is directly caused by Ca^2+^ overload (Jou, 2008; Smaili et al., 2009; Peng and Jou, 2010). VDAC may serve as a junction for the inter-connection of the two signaling molecules, ROS and Ca^2+^. The molecular link between Ca^2+^ and ROS through VDAC is evident, as already discussed. VDAC1 is linked to redox-sensitive ER Ca^2+^-release channel, IP_3_R by GRP-75 chaperone (Szabadkai et al., 2006), indicating that ROS and ER based Ca^2+^ signaling pathways are inter-linked. This inter-connection through VDAC is reported in yeast as well. ATP synthase along with the porin complex regulates Ca^2+^ homeostasis and permeability transition of mitochondria *in vivo* (Niedzwiecka et al., 2018). In lung cancer cells, a member of the Bcl-2 family, Mcl binds with VDAC1 and VDAC2. Disruption in this interaction limits Ca^2+^ uptake, which further inhibits ROS generation and shows VDAC-based Ca^2+^ dependent ROS production (Huang et al., 2014). The generation of oxidative damage in cardiac microvascular endothelial cell injury (CMEC injury) is based on IP_3_R-VDAC-Ca^2+^. Simulation of MAPK/ERK by melatonin inactivates cAMP response element-binding protein (CREB), thus, blocking oxidative stress damage responsible for cardiac dysfunction (Zhu et al., 2018). The closure of VDAC by phosphorothioate increased superoxide in mitochondria because its flux from the inner mitochondrial space to the cytosol is affected. This results in Ca^2+^-induced mitochondrial permeability transition, causing the induced opening of permeability transition pores of high conductance in IMM due to excessive Ca^2+^ uptake (Tikunov et al., 2010). The notable roles of animal VDACs make the effort to pursue plant VDACs for their involvement in stress responses worthy. The research so far is pioneering in showing VDAC as a linking bridge between ROS and Ca^2+^ but we still lag in understanding the initiation of this loop. It is still emerging if mitochondrial Ca^2+^ signal generates a ROS signaling event or flux of ROS via mitochondrial stimulation of Ca^2+^ signaling in cytosol/mitochondria. It is still unclear whether the process is only facilitated by VDACs majorly and independently or does it need a complex to regulate the signaling. We expect studies in the future to depict the cytosolic and mitochondrial Ca^2+^ dynamics in the absence of VDAC in plants that can clarify the involvement of VDAC in the process. The simultaneous imaging of ROS and Ca^2+^ in reference to VDACs and their patterns in real-time also demand attention. Nevertheless, at present, it is an open question that needs to be investigated in plants. We also expect research in the future that can solve the mode of regulation in VDACs in these signaling events. It is a challenging opportunity to elucidate the entire signaling mechanism, including Ca^2+^-ROS homeostasis, in the context of cell biology. The interconnection between pathways and the role of VDACs as the central node as shown in Figure 2 is highly important and needs to be pursued further.

## Conclusion and Future Perspective

Significant knowledge on VDAC has accumulated in recent years. The functional range of VDACs as gating proteins has now expanded. We know the localization of VDACs, their modulation, biochemical regulation and functional implications in animals and, to some extent, in plants. Multi-localization of VDAC on different membranes, yet the difference in their functions, is an example of how nature increases diversity with existing tools in the cell. The multi-localization of VDAC can be predicted as a connecting route for better intra-cellular communication. However, its localization on PM is highly debatable. Different modes of regulation of VDACs make them versatile and hence, explains their ability to regulate and participate in multiple signaling events. The research on biochemical regulation is still limited and extensive intervention is the need of the hour. These channels are well known for molecular transport, and this function also puts them under the spotlight as communicators between cytoplasmic and mitochondrial signaling events. A mutual interplay between Ca^2+^ and ROS is also mediated by VDACs, and hence, they can be a Ca^2+^-ROS connecting link. However, many interesting questions remain to be addressed. At present, the ongoing research on plant VDACs faces many challenges, with burning questions and controversies. Its structure, isoforms in plants, role in apoptosis, relation with PCD and the VDAC interactome are gray areas. The active components extracted from plants can modulate animal VDACs. Their functional significance on plant VDACs will be a great help to further our understanding. Fortunately, the once lagging plant VDACs have emerged as a new arena of research. Based on the studies so far, VDACs have immense potential in therapeutics and the improvement of crops. The ongoing research will further unmask its functional significance, particularly, in plants.

## Author Contributions

GP conceptualized the review. BR and GP wrote the manuscript. BR, PK, SS, MB, and GP reviewed and revised the manuscript. All authors contributed to the article and approved the submitted version.

## Conflict of Interest

The authors declare that the research was conducted in the absence of any commercial or financial relationships that could be construed as a potential conflict of interest.

## Publisher’s Note

All claims expressed in this article are solely those of the authors and do not necessarily represent those of their affiliated organizations, or those of the publisher, the editors and the reviewers. Any product that may be evaluated in this article, or claim that may be made by its manufacturer, is not guaranteed or endorsed by the publisher.
